# High-fat diet-induced brain region-specific phenotypic spectrum of CNS resident microglia

**DOI:** 10.1007/s00401-016-1595-4

**Published:** 2016-07-08

**Authors:** Caroline Baufeld, Anja Osterloh, Stefan Prokop, Kelly R. Miller, Frank L. Heppner

**Affiliations:** 1 Department of Neuropathology, Charité-Universitätsmedizin Berlin, Charitéplatz 1, 10117 Berlin, Germany; 2 Center for Neurologic Diseases, Department of Neurology, Brigham and Women’s Hospital, Harvard Medical School, Boston, MA USA; 3 Department of Pathology and Laboratory Medicine, Hospital of the University of Pennsylvania, Philadelphia, PA USA; 4 Center for Neurodegenerative Disease Research, Department of Pathology and Laboratory Medicine, University of Pennsylvania, Philadelphia, PA USA; 5 Cluster of Excellence, NeuroCure, Charitéplatz 1, 10117 Berlin, Germany; 6 Berlin Institute of Health (BIH), Berlin, Germany

**Keywords:** Microglia, Glia, High-fat diet, Metabolism

## Abstract

Diets high in fat (HFD) are known to cause an immune response in the periphery as well as the central nervous system. In peripheral adipose tissue, this immune response is primarily mediated by macrophages that are recruited to the tissue. Similarly, reactivity of microglia, the innate immune cells of the brain, has been shown to occur in the hypothalamus of mice fed a high-fat diet. To characterize the nature of the microglial response to diets high in fat in a temporal fashion, we studied the phenotypic spectrum of hypothalamic microglia of mice fed high-fat diet for 3 days and 8 weeks by assessing their tissue reaction and inflammatory signature. While we observed a significant increase in Iba1+ myeloid cells and a reaction of GFAP+ astrocytes in the hypothalamus after 8 weeks of HFD feeding, we found the hypothalamic myeloid cell reaction to be limited to endogenous microglia and not mediated by infiltrating myeloid cells. Moreover, obese humans were found to present with signs of hypothalamic gliosis and exacerbated microglia dystrophy, suggesting a targeted microglia response to diet in humans as well. Notably, the glial reaction occurring in the mouse hypothalamus was not accompanied by an increase in pro-inflammatory cytokines, but rather by an anti-inflammatory reaction. Gene expression analyses of isolated microglia not only confirmed this observation, but also revealed a downregulation of microglia genes important for sensing signals in the microenvironment. Finally, we demonstrate that long-term exposure of microglia to HFD in vivo does not impair the cell’s ability to respond to additional stimuli, like lipopolysaccharide. Taken together, our findings support the notion that microglia react to diets high in fat in a region-specific manner in rodents as well as in humans; however, this response changes over time as it is not exclusively pro-inflammatory nor does exposure to HFD prime microglia in the hypothalamus.

**Electronic supplementary material:**

The online version of this article (doi:10.1007/s00401-016-1595-4) contains supplementary material, which is available to authorized users.

## Introduction

Obesity is a disease affecting millions of people worldwide [6, 7]. Past research has focused heavily on the effects of diets high in fat content on peripheral organs, such as adipose tissue, where obesity leads to a chronic low-level inflammation that is central to peripheral metabolic disease [19]. This inflammation is mediated by macrophages, which infiltrate the expanding adipose tissue, and ultimately results in insulin resistance [20, 39]. Recent work exploring the effects of obesity and metabolic disease on the central nervous system (CNS) revealed that the metabolic syndrome, concomitant with type II diabetes, is an important risk factor for neurodegeneration and cognitive dysfunction [8, 22]. Long-term high-fat diet (HFD) leads to brain inflammation [28, 41] and leptin resistance in the hypothalamus [14]. Furthermore, HFD induces endoplasmic reticulum (ER) stress and apoptosis in hypothalamic neurons and inhibits neurogenesis in this region [27, 36]. Microglia, the brain’s intrinsic immune cells, play an essential role in physiological brain functions, including pruning of neuronal synapses and regulation of brain development [13, 31] and respond to disease or injury to the CNS [33, 37, 40]. Fitting with the notion that diets high in fat content are harmful to the brain, reactivity of astrocytes and microglia was observed as early as 3 days after the start of HFD in rats [36]. This response seems to be caused by dietary factors and hormonal changes rather than by increases in body weight itself [17], suggesting that CNS resident glia directly sense nutrient components.

Our aim was to analyze the nature of the microglia reaction to high-fat diet in mice over time and investigate whether the inflammatory response induced by high-fat diet is mediated primarily by CNS resident microglia or rather represents a classical inflammatory disease in which peripheral monocytes invade the brain parenchyma, as is seen in peripheral organs in metabolic disease and classical inflammatory CNS diseases, such as multiple sclerosis. Our analyses reveal a specific reaction of hypothalamic microglia to high-fat diet. Interestingly, this reaction consists of a mixed pro- and anti-inflammatory response in the hypothalamic tissue. Through the use of bone marrow chimeras, we were able to demonstrate that endogenous microglia mediate the hypothalamic response to high-fat diet in the absence of infiltrating monocytes. Finally, analysis of human postmortem brain tissue revealed an effect of body mass index (BMI) on microglia in the hypothalamus when comparing individuals with a BMI greater than 30 to persons with a BMI below 25, implying a specific glial response to diet in humans as well.

## Methods

### Mice

Adult male C57Bl/6 J mice aged 100–120 days were used for all experiments and kept under specific pathogen-free conditions on a 12-h light/dark cycle, and food and water were provided ad libitum. To study the effect of HFD, mice were fed either a HFD (60 % kcal % fat, Research Diets, D12492) or recommended low-fat diet (10 % kcal % fat, Research Diets, D12450B) for different time periods between 3 days and 20 weeks. A detailed description of the different time points and the experiments for which they were used is shown in Table 1. The earlier time points were chosen based on a previous report [36] and extended to 8 and 16–20 weeks to include a time point at which obesity is clearly manifested (Supplementary figure 1). Food intake and body weight were measured once a week (Supplementary figure 1). All animal experiments were performed in accordance with the national animal protection guidelines approved by the regional office for health and social services in Berlin (LaGeSo).

**Table 1:** Cohorts used for mouse experiments

Cohort	Duration of feeding (chow and HFD)	Application	Figures
(1) *n* = 5 (Chow) *n* = 5 (3 days HFD) *n* = 3 (4 weeks HFD) *n* = 7 (8 weeks HFD)	3 days, 4 and 8 weeks	Histology	1
(2) *n* = 7 (Chow) 8 (HFD)	20 weeks	Bone marrow chimeric experiments, BrdU administration	3
(3) *n* = 6–7	3 and 7 days, 4 and 8 weeks	qPCR gene expression analysis	4
(4) *n* = 5 per diet	3 days and 8 weeks	NanoString mRNA analysis	5
(5) *n* = 30 per diet	16 weeks	Blood for plasma stimulation, microglia for LPS stimulation	6 and 7

### Generation of bone marrow chimeras

For the generation of bone marrow chimeras, 1 × 10^7^ bone marrow cells obtained from tibia and femur of Tg(ACTbEGFP)1Osb (GFP) mice (Jackson Laboratories) were injected into the tail vein of C57BL/6 mice exposed to 10 gray whole-body irradiation. Mice were housed in individually ventilated cages and treated with antibiotic (0.01 % Enrofloxacin, Baytril^®^, Bayer Vital) for 4 weeks. To ensure that the HFD feeding had no influence on the engraftment of transplanted GFP bone marrow, HFD feeding was started after another 4 weeks of recovery. Mice were fed with high-fat diet or low-fat chow for a total of 20 weeks. Previous reports demonstrate that peripheral GFP+ blood leukocytes are not affected by this treatment [9].

For the analysis of proliferation of microglia cells upon HFD, animals received a weekly i.p. injection of Bromodeoxyuridine (BrdU) (50 mg/kg), a thymidine analog that integrates into the DNA during replication.

### Microglia sorting and cell culture

For analysis of isolated murine microglia, mice were anesthetized and perfused with phosphate-buffered saline (PBS). Hypothalamus was dissected from the brain and manually dissociated in HBSS buffer. A neural dissociation kit (Miltenyi Biotech) was used to create a single-cell suspension, which was then incubated with anti-CD11b microbeads (Miltenyi Biotech), and CD11b+ cells were isolated using MACS MS columns (Miltenyi Biotech).

Plasma of mice fed HFD or chow for 16 weeks was collected for stimulation of sorted microglia. Sorted microglia cells were plated in a 24-well plate with 5 × 10^5^ cells per well and 3 wells per condition containing DMEM supplemented with 10 % FBS and 50 U/ml PenStrep. The next day, the medium was replaced with medium containing 10 % plasma. After 5 h, this medium was exchanged with fresh medium that was collected for analysis 1 h later.

For stimulation with lipopolysaccharide (LPS), isolated primary hypothalamic microglia were plated in 96-well plates with 5 × 10^4^ cells per well and 3 wells per condition. The next day, cells were stimulated with 1 µg/ml LPS and the medium was collected 24 h later for cytokine analysis.

### Histology

Brains were removed and stored in 4 % paraformaldehyde (PFA) overnight. The next day, PFA was replaced by 30 % sucrose for at least 24 h. Brains were mounted on a platform using freezing media and cut coronally on a cryostat at 30 µm. Sections were stored in cyroprotectant at 4 °C until use. For immunohistochemical and immunofluorescent stainings, sections were washed with PBS and incubated in PBS with 0.3 % Triton X-100 and 10 % goat serum for 1 h at room temperature followed by incubation with primary antibodies: Iba1 (1:500, Wako Chemicals, cat. # 019-19741), GFAP (1:5000, Dako, cat. # Z033401-2), GFP (1:1000, Abcam, cat. # ab290) or anti-BrdU (1:500, AbD Serotec, cat. # MCA2060GA) at 4 °C overnight. Sections were then incubated with a peroxidase-coupled goat anti-rabbit antibody (1:200, Dianova, cat. # 111-035-003) and developed with 3,3′-Diaminobenzidine (DAB) solution. For immunofluorescent staining, sections were incubated with Alexa Fluor^®^ 488 anti-rabbit (1:200, Abcam, cat. # ab150077) or anti-rat-Cy3 (1:200, Dianova, cat. # 712-165-153). Fluorescent sections were imaged using a confocal laser-scanning microscope (Leica).

### Stereological and stereomorphometric analysis

Stereoinvestigator software (MBF Bioscience) was used for the assessment of Iba1+ and GFP+ cells. Cells were counted using the Optical Fractionator method in a total of 8–10 sections per mouse collected at an interval of 6 sections apart, as previously described [5]. For analysis of GFAP staining, pictures of 6–8 sections were analyzed with the CellSense software (Olympus) using the phase analysis tool, as previously described [5].

### Mesoscale

Serum parameters and pro-inflammatory cytokines were measured with Mesoscale [Mouse Metabolic Kit, V-PLEX Plus Proinflammatory Panel 1 (mouse) Kit] according to the manufacturer’s instructions.

### qPCR

For the analysis of gene expression of whole hypothalamic tissue, RNA was isolated using the InviTrap^®^ Spin Tissue RNA Mini (Invitek Inc., Berlin, Germany). 1 μg of RNA was converted to cDNA using the QuantiTect Reverse Transcription Kit (Qiagen). qPCR was carried out using the TaqMan^®^ Fast Universal PCR Master Mix and gene-specific TaqMan^®^ gene expression assays (Life Technologies). qPCR results were analyzed using the delta–delta Ct method and gene expression of the target gene was normalized to that of *Gapdh*, as previously published [5].

### Quantitative NanoString nCounter gene expression analysis

For NanoString nCounter analysis (NanoString Technologies) of gene expression of isolated microglia, RNA of sorted microglia cells was isolated using the PicoPure^®^ RNA Isolation Kit (Life Technologies) according to the manufacturer’s instructions. 10,000 cells per sample were used to measure transcript levels of 42 target and 6 housekeeping genes (Table 2). Measurements were performed at the University Medical Center Goettingen Transcriptome and Genome Analysis Laboratory (Goettingen, Germany). Results were analyzed using nSolver™ analysis software 2.5.

**Table 2:** Accession number and name of genes analyzed using NanoString nCounter

Accession number	Gene name	Accession number	Gene name
NM_019741	Slc2a5	NM_011313	S100a6
NM_009151	Selplg	NM_009115	S100b
NM_178706	Siglech	NM_213659.2	Stat3
NM_008479	Lag3	NM_010548.1	Il10
NM_001164034	Ntf3	NM_008361.3	Il1b
NM_009917.5	CCr5	NM_031168.1	Il6
NM_007651.3	Cd53	NM_007707.2	Socs3
NM_011905.2	Tlr2	NM_011577.1	Tgfb1
NM_021297.2	Tlr4	NM_009367.1	Tgfb2
NM_001042605.1	CD74	NM_009368.2	Tgfb3
NM_011146.1	Pparg	NM_009369.4	Tgfbi
NM_008352.1	Il12b	NM_009370.2	Tgfbr1
NM_031252.1	Il23a	NM_009371.2	Tgfbr2
NM_008625	mrc1	NM_031254.2	Trem2
NM_008689.2	Nfkb	NM_011662.2	Tyrobp
NM_010546.2	Ikbkb	NM_008746	TrkC
NM_010745.2	Ly86	NM_007540	Bdnf
NM_008320.4	Irf8	Housekeeping genes	
NM_001291058.1	CD68	NM_020559.2	Alas1
NM_009987.4	Cx3cr1	NM_026007.4	Eef1g
NM_009142.3	Cx3cl1	NM_008062.2	G6pdx
NM_001111275.1	Igf1	NM_001001303.1	Gapdh
NM_146162.2	Tmem119	NM_010368.1	Gusb
NM_027571.3	P2ry12	NM_013556.2	Hprt
NM_013693.1	Tnf		

### Human tissue

Brain autopsies were performed following written consent for pathological examination according to the law of Berlin. Following routine diagnostic neuropathological examination, the hypothalamus and parts of the frontal cortex were obtained and used for sectioning and immunohistochemical stainings. This procedure was approved by the Charité ethics commission (EA1/019/13). Cases with infectious or inflammatory disease (peripheral or central), psychotropic drug use, history of substance addiction, chronic anti-inflammatory or immunosuppressive therapy, clinically or pathologically symptomatic brain edema, intracerebral hemorrhage, brain irradiation, chemotherapy, hypoxic or ischaemic damage or primary CNS pathology including neurodegenerative disease were excluded from the analysis. The postmortem interval was not considered as central inclusion criterion for this analysis as it has been shown to have no effect on microglia phenotype [24]. Due to the strict exclusion criteria, only 1.6 % of the approximately 600 cases that are collected per year could be used for this analysis. Information about gender, age and BMI of the analyzed cases is given in Table 3.

**Table 3:** Summary of human cases

	Non-obese (BMI < 25)	Obese (BMI ≥ 30)
#	9	12
BMI	23 ± 1.9	36 ± 4.2
Gender	7 m/2 f	9 m/3 f
Age (year)	65 ± 17.2	69 ± 12

Formalin-fixed tissue was placed in 30 % sucrose for at least 1 day and then cut frozen on a cryostat into 50-µm-thick sections, which were stored in cryoprotectant at 4 °C until use. The sections were then stained using the same procedure as for the mouse tissue.

Area covered by Iba1 and GFAP immunoreactivity was analyzed in 10 sections per individual in the hypothalamus as depicted in Supplementary figure 2a. The same sized area was analyzed in the frontal cortex of the same individual and data are displayed as a ratio between the two regions to account for any confounding effects that may modify microglia phenotype. To quantify dystrophic microglia, three stages of dystrophy/cytorrhexis were defined according to Streit et al. [35] and illustrated in Supplementary figure 2b: + beading and partial fragmentation of processes, ++ complete fragmentation of processes while still maintaining cell contours, and +++ scattered fragments with intact nucleus. Ten random images per specimen were taken and dystrophic microglia were displayed as percentage of all cells per image to account for differences in cell number between images/specimen.

### Statistical analysis

Data are expressed as mean ± SEM. Comparisons between two groups were performed using Student’s *t* test. For the comparison of more than two groups, two-way ANOVA with Bonferroni’s post hoc analysis was used.

## Results

### Prolonged HFD exposure leads to gliosis in the mouse hypothalamus

**Fig. 1:**
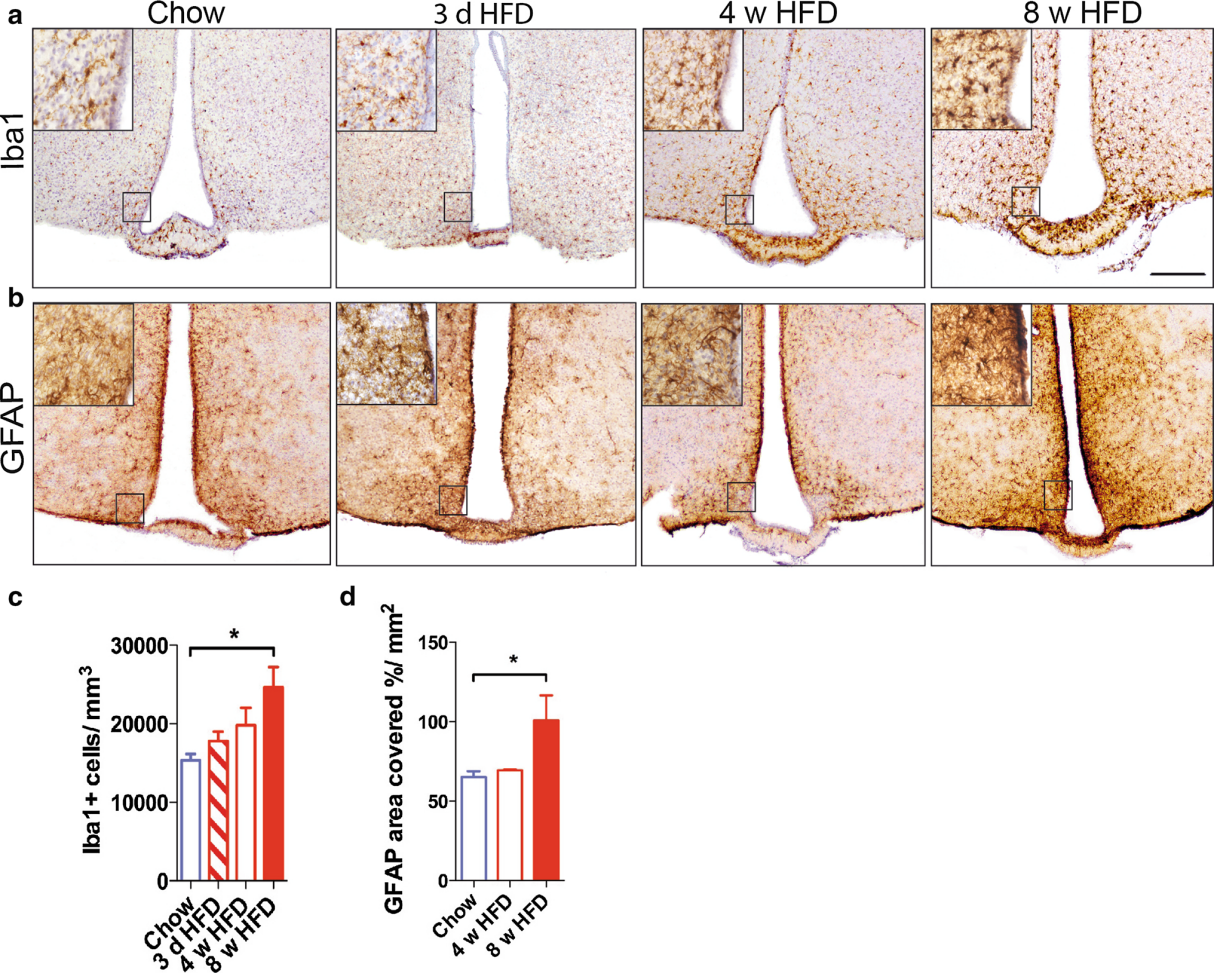
Gliosis in the mouse hypothalamus in response to HFD. **a** Iba1 and **b** GFAP immunoreactivity in the hypothalamus of HFD- and chow-fed wild-type mice. **c** Quantification of Iba1-positive cells in the hypothalamus of HFD- vs. chow-fed wild-type mice. *n* = 5 (Chow), *n* = 5 (3 d HFD), *n* = 3 (4 w HFD), *n* = 7 (8 w HFD). **d** Quantification of area covered by GFAP-positive cells in the hypothalamus of HFD- vs. chow-fed wild-type mice. *n* = 5 (Chow), *n* = 3 (4 w HFD), *n* = 3 (8 w HFD). Statistical analyses: *P < 0.05 based on Kruskal–Wallis test with Dunn’s Multiple Comparison Post-Test. *Scale bar* 200 µm. Data represent mean ± SEM. *HFD* high-fat diet, *d* days, *w* weeks

To confirm reports of pro-inflammatory responses of microglia to HFD [36] and expand upon previous studies, we assessed the effects of variable durations of HFD feeding on microglia and astrocyte quantity and morphology in the hypothalamus. To this end, we used the microglia/myeloid cell-specific marker Iba1 and the astrocyte marker GFAP for histological analysis. We did not detect morphological alterations or an increase in microglia cell number after 3 days of HFD in mice (Fig. 1a, c). After 4 weeks of HFD, we detected a slight trend towards a higher number of myeloid cells in the hypothalamus, while after 8 weeks of HFD, a time point when the mice exhibit a 50 % increase in body weight and elevated serum leptin level (Supplementary figure 1a–d), the number of Iba1+ cells was significantly higher than in chow-fed mice (Fig. 1a, c). Similarly, the area covered by GFAP-positive astrocytes was also significantly increased after 8 weeks of HFD (Fig. 1b, d).

### Gliosis is evident in the hypothalamus of humans with BMI > 30

**Fig. 2:**
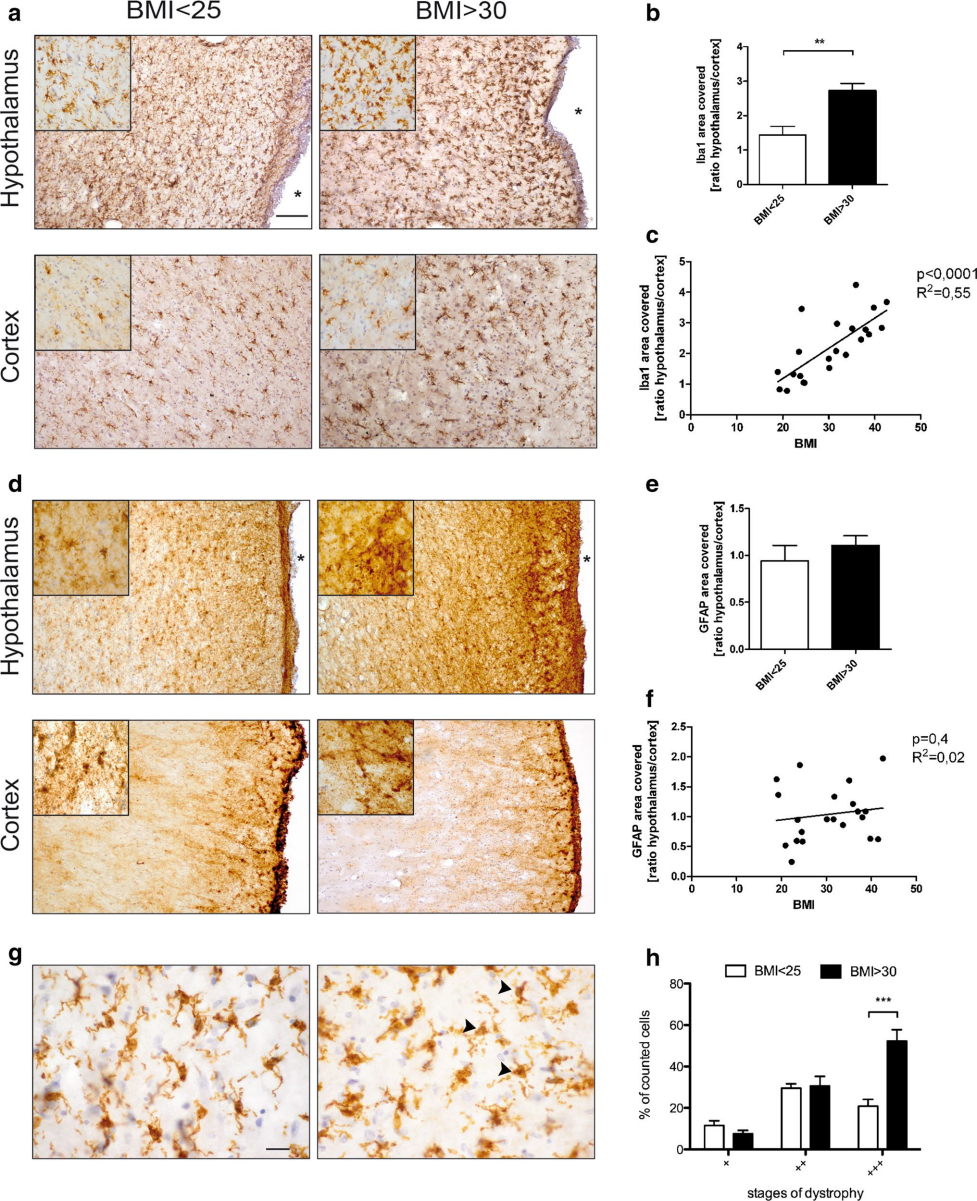
Gliosis in the hypothalamus of human individuals with BMI > 30. **a** Iba1 immunoreactivity in the hypothalamus (*upper panel*) and cortex (*lower panel*) of individuals with BMI < 25 vs. BMI > 30. **b** Ratio of area covered by Iba1 immunoreactivity in the hypothalamus versus cortex of individuals with BMI < 25 vs. BMI > 30. *Scale bar* 100 µm. **c** Correlation of area covered by Iba1 immunoreactivity in the hypothalamus (normalized to the respective individual cortical Iba1 immunoreactivity) to BMI. **d** GFAP immunoreactivity in the hypothalamus (*upper panel*) and cortex (*lower panel*) of individuals with BMI < 25 vs. BMI > 30. *Scale bar* 100 µm. **e** Ratio of GFAP-covered area in the hypothalamus versus cortex of individuals with BMI < 25 vs. BMI > 30. **f** Correlation of area covered by GFAP immunoreactivity in the hypothalamus (normalized to the respective individual cortical GFAP immunoreactivity) to BMI. *n* = 9 (BMI < 25), 12 (BMI > 30). **g** Close-up of Iba1 + cells in the hypothalamus of individuals with BMI < 25 (*left panel*) vs. BMI > 30 (*right panel*). *Arrowheads* depict late stages of microglia dystrophy. *Scale bar* 20 µm. **h** Quantification of different stages of microglial dystrophy in the hypothalamus of individuals with BMI < 25 and BMI > 30. *n* = 9 (BMI < 25), 12 (BMI > 30). Statistical analyses: **b** **p < 0.01 based on unpaired student’s *t* test. **h** ***p < 0.001 based on two-way ANOVA with Bonferroni post hoc test; interaction: *F* (1, 54) = 9.162, *p* = 0.0038. Data represent mean ± SEM. *Asterisk* marks the third ventricle. *BMI* body mass index

To determine if a similar glial response occurs in the brains of obese humans as was observed in the brains of HFD-fed mice, we analyzed postmortem hypothalamic and cortical brain tissue from normal weight individuals with BMI < 25 and obese individuals with BMI > 30, which was collected under very strict exclusion criteria (for a detailed list see methods section). Histological analysis of the brains of obese individuals revealed Iba1+ cells with aberrant morphology indicative of microglia dystrophy, including enlarged cell bodies, shortened processes and apparent cytorrhexis, respectively, exclusively adjacent to the third ventricle in the hypothalamic area (Fig. 2a, upper right, and Fig. 2g, right panel), whereas Iba1 + cells in the cortex exhibited an inconspicuous morphology characterized by small cell bodies and delicate, ramified processes both in obese and non-obese subjects (Fig. 2a, bottom panels).

To quantify the degree of microglia changes, the area covered by Iba1+ cell bodies in the area of the arcuate nucleus of the hypothalamus (Supplementary figure 2a) was normalized to that of the cortical Iba1 area covered for each individual. Not only was the ratio of hypothalamic/cortical Iba1 covered area significantly increased in individuals with BMI > 30, but correlated significantly overall with the BMI (Fig. 2b, c) revealing that microglia changes are associated with increased BMI. Quantification of microglia dystrophy revealed that while most microglia in the analyzed region of the hypothalamus displayed signs of dystrophy in all individuals (Fig. 2g), significantly more microglia displayed the most severe signs of dystrophy/cytorrhexis in the group of obese individuals (Fig. 2h). The astrocytic response was not as robust as the microglial response to BMI, as the observed increase in the ratio of hypothalamic to cortical GFAP did not differ significantly between individuals with BMI < 25 compared to those with BMI > 30 (Fig. 2d–f).

### Peripheral myeloid cells do not contribute to the CNS immune response to HFD

**Fig. 3:**
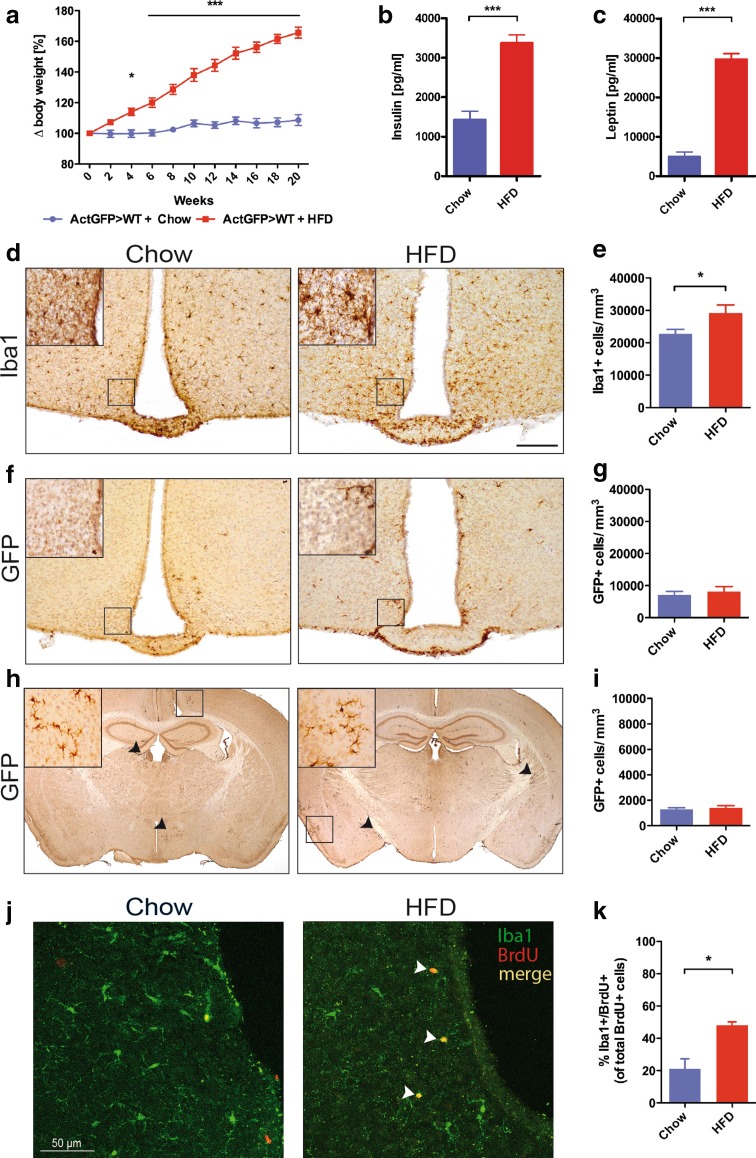
HFD leads to proliferation of endogenous microglia in the hypothalamus. **a** Body weight development of Actin-GFP bone marrow chimeric mice fed either HFD or chow for 20 weeks. **b** Serum insulin and **c** serum leptin levels of Actin-GFP bone marrow chimeric mice fed either HFD or chow for 20 weeks. **d** Iba1 immunoreactivity of myeloid cells in the hypothalamus of Actin-GFP bone marrow chimeras fed HFD or chow for 20 weeks. **e** Quantification of Iba1-positive cells in the hypothalamus of Actin-GFP bone marrow chimeras fed HFD or chow for 20 weeks. **f** GFP immunoreactivity in the hypothalamus of Actin-GFP bone marrow chimeras fed HFD or chow for 20 weeks. **g** Quantification of GFP-positive cells in the hypothalamus of Actin-GFP bone marrow chimeras fed HFD or chow for 20 weeks. **h** GFP immunoreactivity in the whole brain of Actin-GFP bone marrow chimeras fed HFD or chow for 20 weeks. **i** Quantification of GFP-positive cells in the whole brain of Actin-GFP bone marrow chimeras fed HFD or chow for 20 weeks. *Scale bar* 200 µm. **j** Iba1 (*green*)/BrdU (*red*)-immunoreactivity in chow- and HFD-fed Actin-GFP bone marrow chimeras. **k** % of Iba1 +/BrdU + cells of all BrdU + cells in the hypothalamus of chow- and HFD-fed Actin-GFP bone marrow chimeras. *n* = 7 Chow, *n* = 8 HFD. Statistical analyses: **a** *p < 0.05 and ***p < 0.001 based on two-way ANOVA with Bonferroni post hoc test; interactions: *F* (1, 143) = 653.21, *p* < 0.0001; **b**, **c**, **e**, **k** *p < 0.05 and ***p < 0.001, respectively, based on unpaired student’s *t* test. Data represent mean ± SEM. *HFD* high-fat diet

Peripheral macrophages infiltrate the adipose tissue and induce inflammation as a result of HFD. To determine the source of the increased number of Iba1+ cells in the hypothalamus, we generated bone marrow chimeric mice harboring actin-GFP bone marrow. To ensure a complete bone marrow engraftment, we waited 8 weeks after the transplantation of GFP bone marrow and then fed the mice with HFD or chow for 20 weeks. This way we were able to discriminate between the endogenous (GFP negative) and the infiltrating peripheral (GFP positive) myeloid cells in the brain. After 20 weeks of HFD, the mice exhibited significant weight gain equivalent to approximately 60 % of their starting body weight, whereas the weight of chow-fed mice only increased by 10 % (Fig. 3a). In addition, HFD-fed bone marrow chimeras exhibited elevated serum insulin and leptin levels, which are associated with an obesogenic state [12, 16] (Fig. 3b, c).

When analyzing the number of myeloid cells in the brain, we found significantly more Iba1+ cells in the hypothalamus of chimeric animals fed HFD (Fig. 3d, e), confirming our earlier findings (Fig. 1a, c). In contrast, there was no quantitative difference in the number of peripherally derived GFP+ myeloid cells in the hypothalamus of mice fed HFD compared to chow (Fig. 3g), which is also evident in histological images (Fig. 3f). Moreover, analysis of the total number of GFP+ cells throughout the whole brain did not reveal a significant difference in the number of CNS-infiltrating macrophages between HFD- and chow-fed bone marrow chimeric mice (Fig. 3h, i). Therefore, these results demonstrate that in our experimental setup, infiltrating peripheral cells do not account for the increase in hypothalamic Iba1+ cells in mice fed HFD (Fig. 3e). Hence, we next assessed proliferation of endogenous microglia upon HFD by weekly pulsing of BrdU, which integrates into the DNA during replication. Quantification of Iba1/BrdU double-positive cells revealed a higher percentage of proliferating microglia (% Iba1+ cells of total BrdU + cells) in the hypothalamus of HFD-fed mice (Fig. 3j, k), thus confirming a specific response of resident microglia to HFD and identifying the specific cellular origin of the increase in microglia number at this time point.

### Prolonged HFD exposure reverses acute hypothalamic pro-inflammatory responses

After excluding a contribution of peripheral macrophages to the hypothalamic response to HFD, we aimed to analyze the microglia response to prolonged HFD in more detail. Results of previous studies hinted towards an early mRNA level proinflammatory response to HFD in the rat hypothalamus after 3 days, which fully manifests after 4 weeks of HFD feeding [36].

**Fig. 4:**
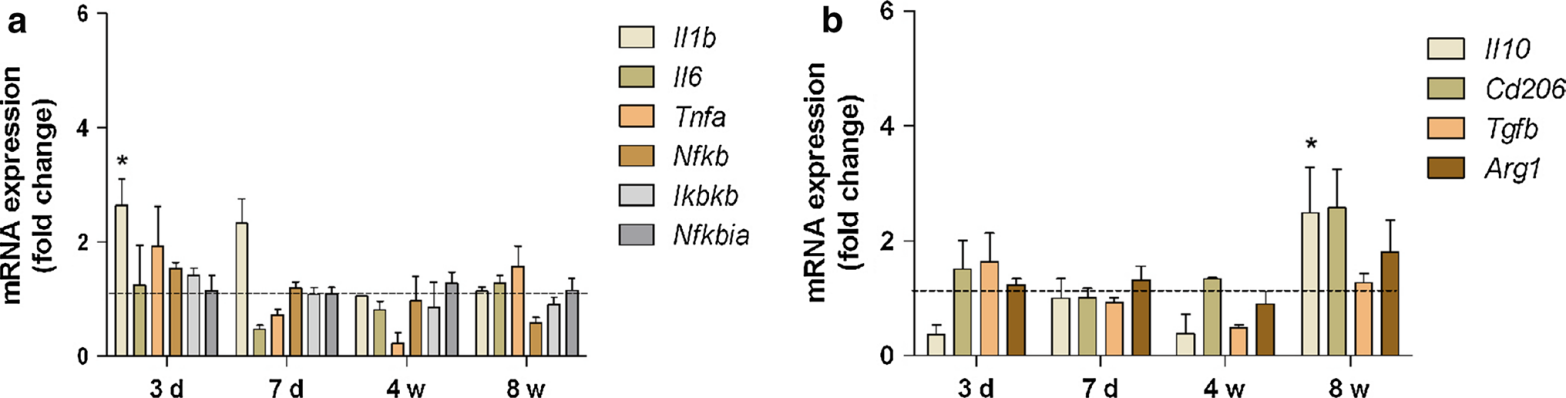
Pro- and anti-inflammatory gene expression in the hypothalamus of HFD-fed mice. **a** Pro- and **b** anti-inflammatory cytokine gene expression in the mouse hypothalamus in response to HFD for 3 days, 7 days, 4 and 8 weeks. *n* = 6–7 per group. Statistical analyses: *P < 0.05 based on two-way ANOVA with Bonferroni post hoc test. Interactions: **a** *F* (3, 103) = 4.04, *p* = 0.0092; **b** *F* (3, 67) = 5.43, *p* = 0.0021. Data represent mean ± SEM. *HFD* high-fat diet, *d* days, *w* weeks

Our analysis of mRNA from whole hypothalamic tissue confirmed a selective upregulation of the pro-inflammatory cytokine interleukin-1b (*Il1b*) after 3 days of HFD (Fig. 4a); however, no overall elevation in pro-inflammatory cytokine mRNA levels was observed after either 4 weeks or even 8 weeks of HFD feeding. In contrast, changes in anti-inflammatory molecules IL-10 (*Il10*), CD206 (*Cd206*) and Arginase1 (*Arg1*) were evident (Fig. 4b), revealing a significant elevation in *Il*-*10*.

### Gene expression profile of isolated hypothalamic microglia corroborates anti-inflammatory profile in response to 8-week HFD feeding

**Fig. 5:**
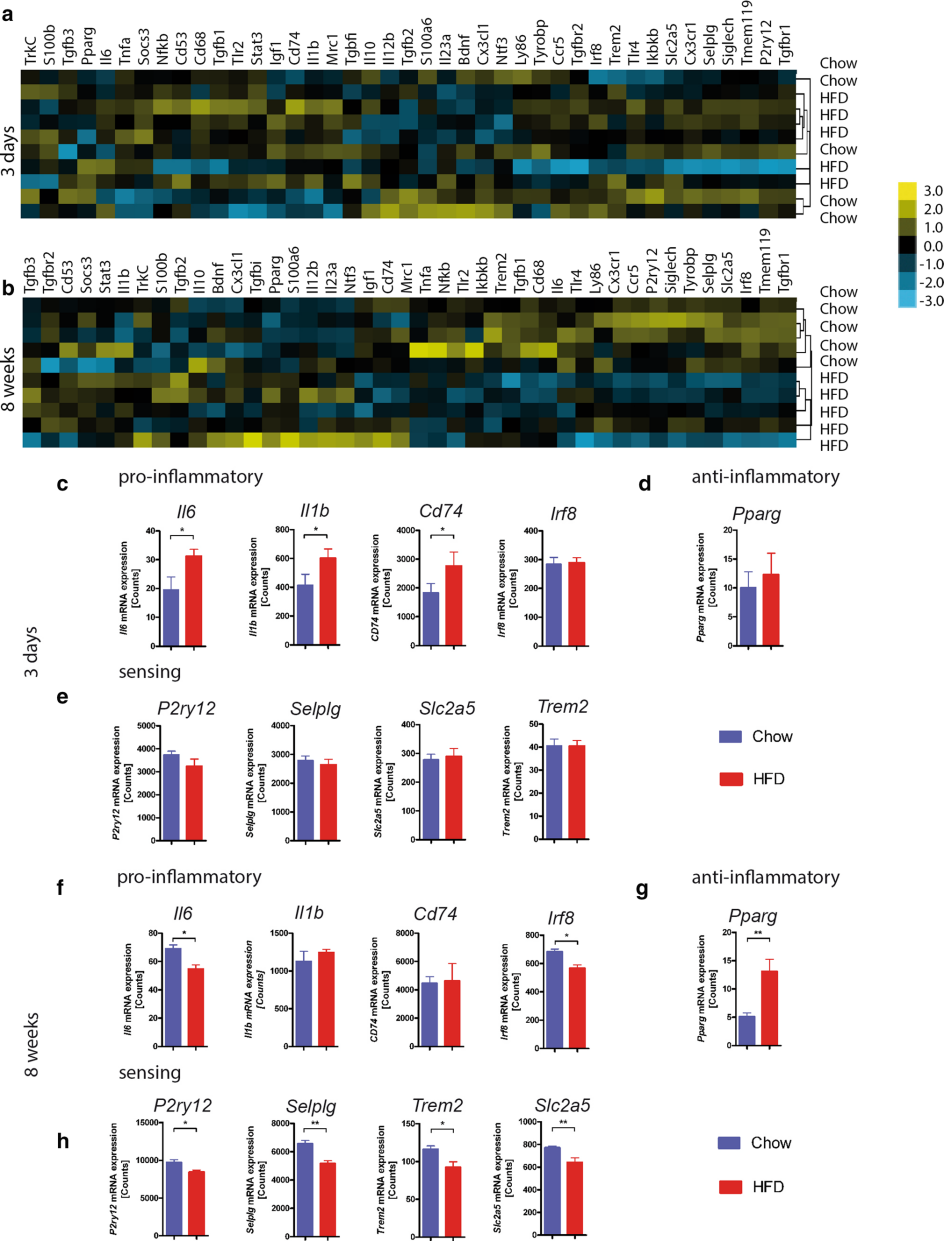
Quantitative gene expression analysis corroborates an anti-inflammatory signature of isolated microglia in response to prolonged HFD. Heat map showing non-biased clustering of normalized gene expression of microglia isolated from the hypothalamus of mice fed HFD or chow for **a** 3 days or **b** 8 weeks. mRNA counts of **c** pro-inflammatory genes (*Il6*, *Il1b*, *Cd74* and *Irf8*), **d** *Pparg* and **e** microglia-specific sensing genes (*P2ry12*, *Selplg*, *Slc2a5* and *Trem2*) in isolated hypothalamic microglia from mice fed HFD or chow for 3 days. mRNA counts of **f** pro-inflammatory genes (*Il6*, *Il1b*, *Cd74* and *Irf8*), **g** *Pparg* and **h** microglia-specific sensing genes (*P2ry12*, *Selplg*, *Slc2a5* and *Trem2*) in isolated hypothalamic microglia from mice fed HFD or chow for 8 weeks. *n* = 5 per group. Statistical analyses: *P > 0.05, **P > 0.01 based on unpaired student’s *t* test. Data represent mean ± SEM

The effects of HFD upon hypothalamic cytokine expression thus far have only been assessed in whole hypothalamic tissue within the framework of this study as well as in all studies published in the literature. Therefore, we sought to specifically assess the microglia reaction to diet using a broader panel of genes than previously employed. For this purpose, hypothalamic microglia isolated from mice fed chow or HFD for 3 days and 8 weeks were analyzed exclusively to quantify the gene expression of 42 selected genes using the NanoString nCounter^®^ Technology. Targets for this analysis included typical inflammatory genes known to be involved in either driving inflammation as pro-inflammatory mediators (e.g. *Il1b*, *Il6*) or attenuating or preventing protracted inflammation (e.g. *Il10*, *Pparg*) as well as genes that have been shown to be specifically enriched in resident microglia cells and involved in their sensing of endogenous and exogenous signals [10, 18] (e.g. *P2ry12*, *Selplg*, *Slc2a5*, *Trem2*) (for complete list see Table 2). Hierarchical clustering of the normalized gene expression shows that after 3 days, the expression profile of microglia from mice fed HFD is not distinct from microglia isolated from chow-fed mice (Fig. 5a). In contrast, 8 weeks of HFD induced a change in the microglial gene expression pattern that is clearly distinguishable from that of microglia isolated from chow-fed mice (Fig. 5b). Analysis of mRNA counts of microglia from mice experiencing HFD for 3 days compared to chow-fed mice confirms our previous findings (Fig. 4a), namely that short-term exposure to a diet high in fat induced increased gene expression of IL-1β (*Il1b*), and expanded on those findings to reveal a general pro-inflammatory reaction with increased gene expression of IL-6 (*Il6*) and CD74 (*Cd74*), a factor involved in MHCII transport and formation as well (Fig. 5c). After 8 weeks of HFD, the gene expression of pro-inflammatory molecules, such as IL-6 or IRF-8, a regulator of microglial reactivity [26], as well as microglia-specific ‘sensing’ genes, including *P2ry12*, *Selplg*, *Slc2a5*, *Trem2*, was reduced (Fig. 5f, h). Moreover, *Pparg* expression, which is associated with anti-inflammatory effects when activated [11], was increased in microglia from HFD-fed mice compared to microglia from chow-fed mice (Fig. 5g).

### Plasma of HFD-fed mice does not induce cytokine production in isolated adult microglia

**Fig. 6:**
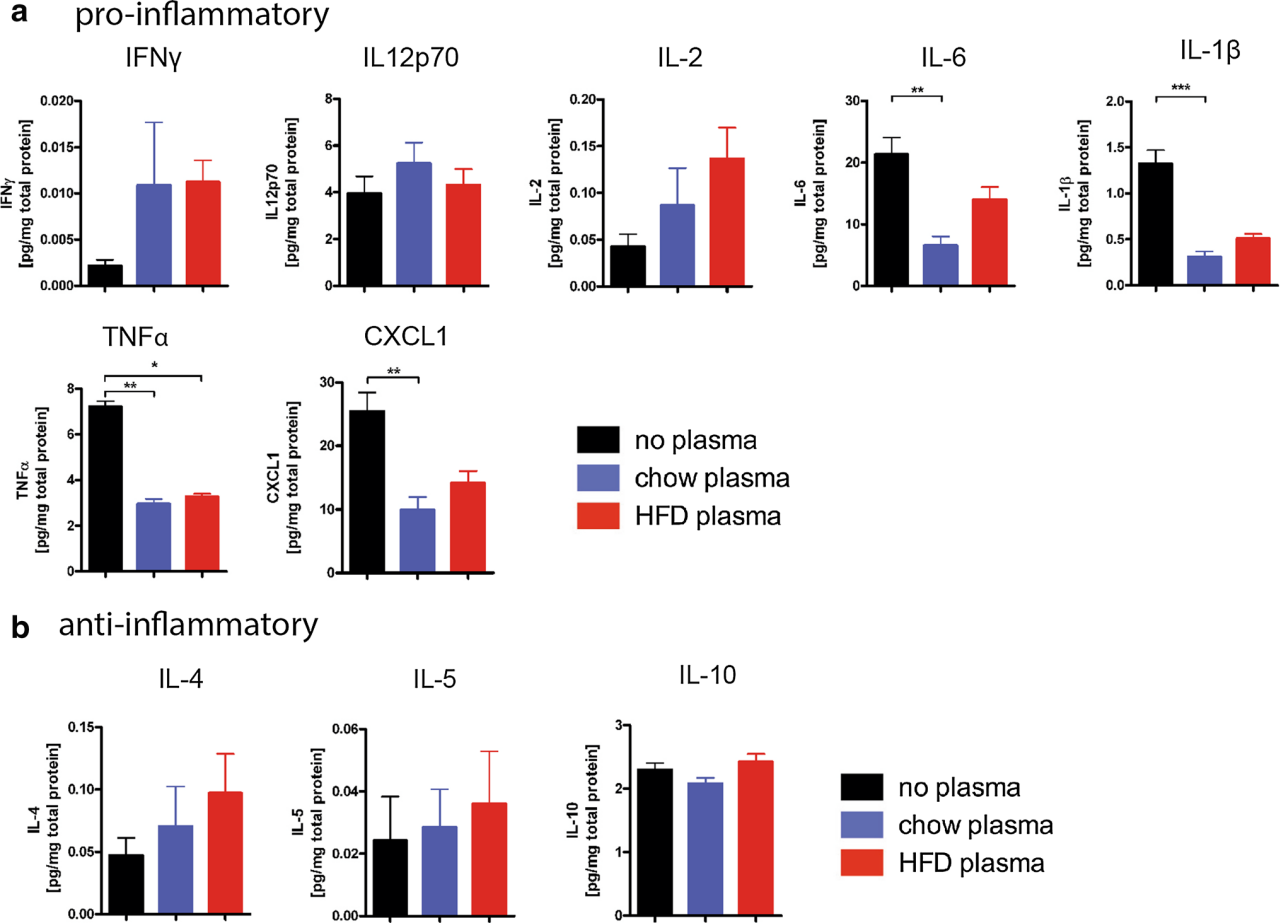
Plasma of HFD-fed mice does not stimulate cytokine production in isolated adult microglia. Protein level of **a** pro-inflammatory factors (IFNγ, IL12p70, IL-2, IL-6, IL-1β, TNFα and CXCL1) and **b** anti-inflammatory factors (IL-4, IL-5, IL-10) in the supernatant of isolated microglia stimulated with 10 % plasma of mice fed HFD or chow for 16 weeks or no plasma as control. *n* = 3 independent experiments. Statistical analyses: *P < 0.05, **P < 0.01, ***P < 0.001 based on Kruskal–Wallis test with Dunn’s Multiple Comparison Post-Test. Data represent mean ± SEM. *HFD* high-fat diet

High-fat diet feeding leads to an increase in hormones (e.g. insulin and leptin) and cytokines in the blood that may be sensed by neurons and microglia in the hypothalamus due to their proximity to the median eminence, a circumventricular organ. To determine if microglia reactivity in the hypothalamus of mice fed a high-fat diet is the result of a primary glial reaction to blood components that may in turn lead to secondary neuronal effects or rather a secondary reaction to altered neuronal homeostasis, we stimulated isolated adult microglia with the plasma of mice that were fed HFD or chow for 16 weeks (Fig. 6). Analysis of cytokines in the microglia-conditioned medium revealed that there was no change in pro- (Fig. 6a) or anti-inflammatory (Fig. 6b) cytokine production in microglia that were stimulated with serum of HFD-fed mice compared to chow serum. This reveals that direct exposure to plasma components from HFD-fed mice does not induce acute pro-inflammatory microglia cytokine responses—at least under these experimental conditions. However, IL-6, IL-1β, CXCL1 and TNFα were reduced in both plasma-treated groups compared to cells without plasma stimulation (Fig. 6a), most likely representing a general reaction to blood plasma factors.

### Microglia exposed to chronic HFD react normally to LPS stimulation

**Fig. 7:**
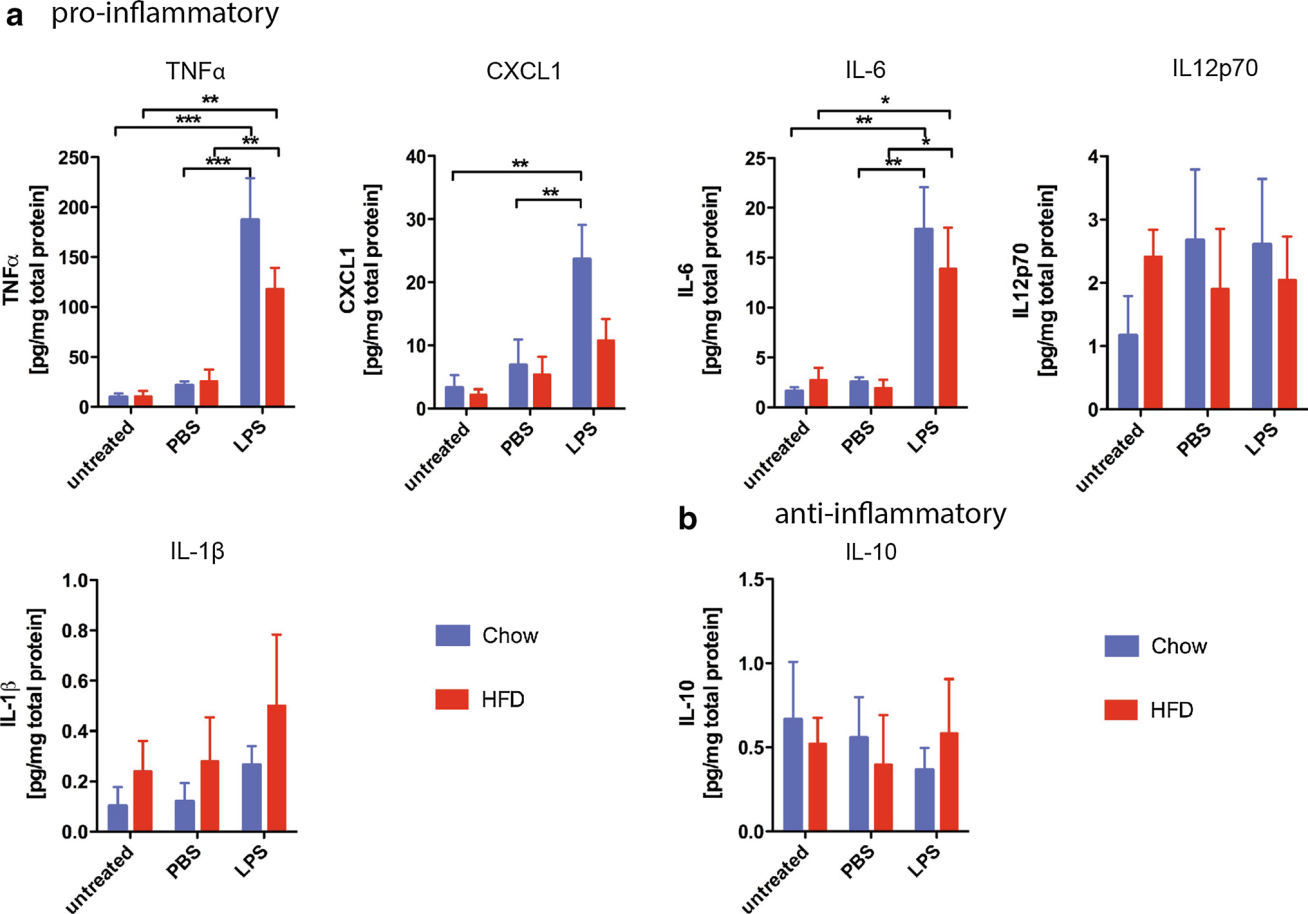
Microglia exposed to chronic HFD ex vivo react normally to LPS stimulation. Protein level of **a** pro-inflammatory factors (TNFα, CXCL1, IL-6, IL12p70 and IL-1 β) and **b** anti-inflammatory IL-10 in the supernatant of untreated, PBS- and LPS-stimulated isolated adult hypothalamic microglia of mice fed HFD or chow for 16 weeks. *n* = 3 independent experiments. Statistical analyses: *P < 0.05, **P < 0.01, ***P < 0.001 based on Two-Way ANOVA with Bonferroni’s Multiple Comparison Post-Test. Interactions: TNFα) *F* (2, 18) = 30.37, *p* < 0.0001; CXCL1) *F* (2, 18) = 9.69, *p* = 0.0014; IL-6) *F* (2, 18) = 16.57, *p* < 0.0001. Data represent mean ± SEM. *LPS* lipopolysaccharide, *HFD* high-fat diet

Microglia priming is a phenomenon known to occur in aging and neurodegenerative diseases [30, 32]. Microglia that are primed are more susceptible to a second inflammatory stimulus and react with an increased production of inflammatory cytokines. A common stimulus used to analyze the microglia inflammatory reaction is lipopolysaccharide (LPS). To assess whether chronic high-fat diet primes microglia or impairs their reactivity to subsequent stimuli, cells were isolated from the hypothalamus of mice fed high-fat diet or chow for 16 weeks, acutely cultured and stimulated with LPS (Fig. 7). Analysis of the basal cytokine production revealed no difference in cytokine expression between microglia of mice fed high-fat diet compared to microglia from chow-fed mice. Furthermore, stimulation with PBS as a control did not cause any changes in the cytokine expression of microglia derived from HFD- or chow-fed mice. On the other hand, LPS stimulation induced a significant increase in TNFα, CXCL1 and IL-6 in microglia independent of the diet to which they were previously exposed (Fig. 7a). IL12p70, IL-1β and IL-10 production was not changed by LPS stimulation (Fig. 7a, b). These results demonstrate that microglia are neither primed by high-fat diet nor impaired in their reaction to strong stimuli like LPS.

## Discussion

Recent studies have reported an early increase in the number of microglia cells in the hypothalamus upon HFD that is accompanied by an upregulation in the expression of markers of microglia activation [17, 36]. In the present study, we were able to confirm an increase in the amount of microglia and astrocytes, but only after 8 weeks of HFD feeding. Furthermore, we aimed to investigate whether changes in the glial populations can also be seen in obese humans, as only radiologic evidence of gliosis in the hypothalamus of obese individuals existed until present [36]. Our analyses demonstrate that glial cells in the hypothalamus (but not in the cortex) of overweight patients are altered, which is more pronounced in microglia cells, but also slightly apparent, though not statistically significant, in the astrocyte population (Fig. 2). Importantly, we saw that not only were microglial changes evident in individuals with BMI > 30, but the degree of microglia alterations correlated significantly with the BMI. Furthermore, analysis of microglia dystrophy in the hypothalamic region revealed that although dystrophic changes were apparent in microglia of all individuals, likely owing to advancing age [24], morphological dystrophy is exacerbated in individuals with BMI > 30. A similar observation has been made in the context of Alzheimer’s disease [33, 34], suggesting that obesity serves to contribute to enhanced microglia stress and potential dysfunction.

Inflammatory brain diseases, such as experimental autoimmune encephalomyelitis (EAE) or stroke, are accompanied by an infiltration of peripherally derived myeloid cells into the brain [1, 23]. When addressing whether similar cellular processes occur in the context of obesity, we found no significant influx of peripheral macrophages into the hypothalamus of GFP^+^-harboring bone marrow chimeric mice after 20 weeks of HFD, in contrast to an earlier report [9]. This difference from the latter study may be due to slightly differing experimental time courses and/or to the fact that the previously published study used FACS analysis to quantify infiltrating cells, which may have resulted in the inclusion of different myeloid subsets (besides resident microglia) such as meningeal or perivascular macrophages. Our finding of a lack of a substantial CNS recruitment of peripheral myeloid cells in HFD-fed mice is further supported by the fact that we were unable to detect elevated levels of the major macrophage-attracting chemokine CCL2 in the hypothalamus (Supplementary figure 1f), which is typically present in situations of myeloid cell recruitment to the CNS [25, 38]. Based on the increase in Iba1/BrdU double-positive cells, we conclude that proliferation of endogenous microglia accounts for the increased myeloid cell numbers in the hypothalamus of HFD-fed mice.

Recent studies have reported increased hypothalamic cytokine gene expression upon HFD that is accompanied by an increase in the expression of markers of microglia activation [17, 36], implying a potentially detrimental microglia response to HFD and obesity. In line with these studies, we also detected an inflammatory response in the hypothalamus only 3 days after initiating HFD. Importantly, however, this response appears to represent an acute reaction to diet in our experimental setting as an elevation in pro-inflammatory cytokine expression was not evident after 4 or 8 weeks of HFD feeding (Fig. 4a). Our findings are consistent with another study using the same HFD formulation in which the authors also failed to observe a pro-inflammatory reaction on either the mRNA or protein level after 8 weeks of feeding [4].

In contrast to the expected pro-inflammatory reaction, we detected an increase in anti-inflammatory molecules in the hypothalamus of mice fed HFD for 8 weeks, suggesting that at this time point, microglia switched from a rather pro-inflammatory to an anti-inflammatory phenotype. This type of temporal, plastic microglia reaction to stimuli has also been reported in other, comparable settings: while acute stimulation with LPS can induce a pro-inflammatory response, chronic LPS stimulation can lead to an increase in anti-inflammatory markers [2, 3]. Furthermore, in contrast to our study, previous analyses have focused exclusively on whole hypothalamic tissue making it impossible to exactly determine the cellular source of secreted factors. Since small, but physiologically relevant changes in cellular genes may not be detected in whole tissue preparations, we decided to analyze the genetic profile of hypothalamic microglia exclusively by acutely isolating them from mice fed HFD or chow at different time points. Our results confirm that microglia indeed produce pro-inflammatory factors after short-term exposure to HFD, but adopt a rather anti-inflammatory phenotype in response to prolonged HFD feeding. The genes we chose to study were previously identified as factors that are important for microglial sensing of endogenous and exogenous signals [18]. Such factors were shown to decrease with aging to presumably ‘tone down’ the otherwise detrimental microglia reaction and prevent chronic activation. Similarly, we detected a downregulation of these microglia ‘sensome’ genes (*P2ry12*, *Selplg*, *Slc2a5*, *Trem2*), which suggests that microglia actively regulate their response to prolonged HFD in such a way as to avoid bystander damage. These findings raise the possibility that neuronal stress and apoptosis occurring in response to HFD [29, 36] are not likely due to a neurotoxic, pro-inflammatory microglia response, as has been speculated. Conversely, this subdued microglia phenotype may serve as a protective mechanism in response to insult that is aimed at preserving neuronal homeostasis. These data are in line with recent findings of elevated anti-inflammatory gene expression in human adipose tissue [15], suggesting analogous responses in the CNS and peripheral organs.

Moreover, analysis of cytokine expression of isolated adult microglia directly stimulated with the plasma of HFD-fed mice indicates that there is no excessive reaction of microglia cells to factors present in the plasma of HFD-consuming, overweight animals. This finding is in contrast to previous studies that have hinted at the possibility that blood-borne factors from HFD-fed mice influence the microglia reaction [17, 21]. Notably, in contrast to our study, the published data are based on cultured neonatal microglia, which have been shown to be genetically and functionally distinct from adult microglia [10] and are, therefore, likely to elicit a differential response to stimuli. Furthermore, microglia chronically exposed to HFD in mice are not primed nor significantly impaired in their response to additional stimuli like LPS and thus appear to be functional and well able to react to changes in their surroundings.

Taken together, our results demonstrate that high-fat diet specifically stimulates endogenous microglia in the hypothalamus and that the microglial response is not exclusively pro-inflammatory. Prolonged exposure to HFD results in an alternate microglia profile represented by a downregulation of microglia-specific genes involved in sensing microenvironmental alterations, likely serving to counterbalance earlier pro-inflammatory changes. This type of response appears to be a typical reaction of microglia to chronic diseases or insults and implies that diets high in fat represent a chronic challenge to CNS and microglia homeostasis. Since specific glial changes can also be found in the hypothalamus (but not the cortex) of obese humans, the glial reaction to diet needs to be studied in further detail, to ultimately determine how it might influence neuronal activity and overall metabolic health.

## Electronic supplementary material

Below is the link to the electronic supplementary material.

Body weight, food intake and metabolic markers of mice fed HFD along different paradigms. (a) Body weight development and (b) cumulative food intake of C57Bl/6 J mice fed either HFD or chow for 8 weeks. n = 8. Serum insulin and leptin level of C57Bl/6 J mice fed either HFD or chow for (c) 4 weeks or (d) 8 weeks. n = 8. (e) Cumulative food intake of Actin-GFP bone marrow chimeric mice fed either HFD or chow for 20 weeks. n = 7 chow, n = 8 HFD. (f) *Ccl2* mRNA expression in C57Bl/6 J mice fed either HFD or chow for 8 weeks. n = 6-7. Statistical analyses: *P < 0.05, **P < 0.01, ***P < 0.001 based on Two-Way ANOVA with Bonferroni’s multiple comparison post-test. Interactions: a) F (1,90) = 256.94, p < 0.0001; b) F (1,80) = 641.62, p < 0.0001; c) F (1,28) = 3.99, p = 0.05; d) F (1,26) = 33.23, p < 0.0001; e) F (1,130) = 245.44, p < 0.0001. Data represent mean ± s.e.m. HFD, high fat diet (JPEG 1714 kb)

Analysis of human hypothalamus. (a) Illustration of a coronal section of the human brain containing a macroscopic picture of the hypothalamus and neighboring structures. Square indicates the area analyzed for Iba1 and GFAP immunoreactivity. (b) Exemplary images of the three morphological presentations of microglia dystrophy which were defined according to Streit et al. [35]: + beading and partial fragmentation of processes, ++ complete fragmentation of processes while still maintaining cell contours and +++ scattered fragments with intact nucleus (TIFF 10523 kb)
