# Supplementary figures and images for: High-fat diet-induced brain region-specific phenotypic spectrum of CNS resident microglia
Source: Acta Neuropathol. 2016 Jul 8;132:361–75. doi: 10.1007/s00401-016-1595-4 (PMC4992033; doi:10.1007/s00401-016-1595-4)

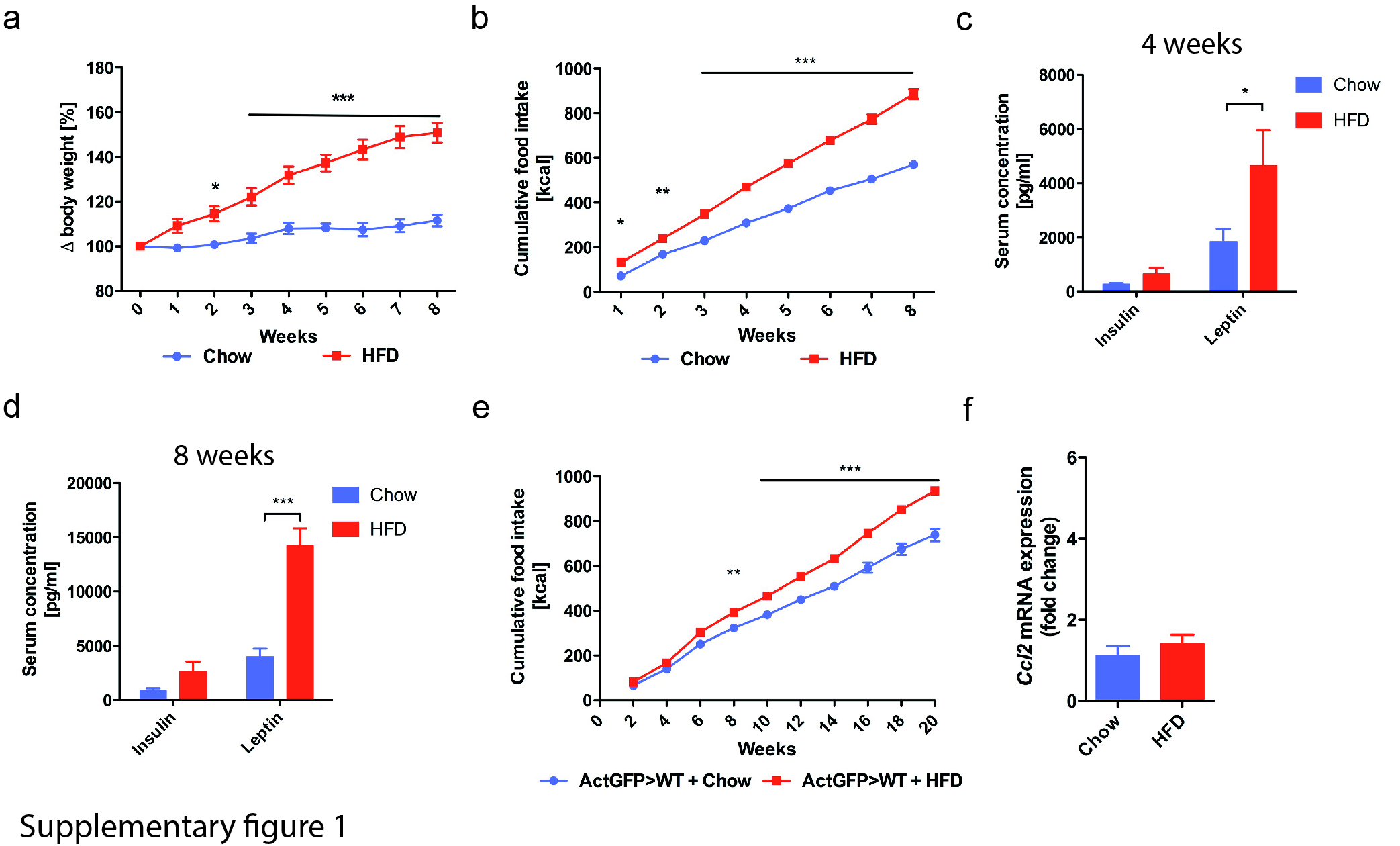

Supplement: Supplementary file 1 — Body weight, food intake and metabolic markers of mice fed HFD along different paradigms. (a) Body weight development and (b) cumulative food intake of C57Bl/6 J mice fed either HFD or chow for 8 weeks. n = 8. Serum insulin and leptin level of C57Bl/6 J mice fed either HFD or chow for (c) 4 weeks or (d) 8 weeks. n = 8. (e) Cumulative food intake of Actin-GFP bone marrow chimeric mice fed either HFD or chow for 20 weeks. n = 7 chow, n = 8 HFD. (f) Ccl2 mRNA expression in C57Bl/6 J mice fed either HFD or chow for 8 weeks. n = 6-7. Statistical analyses: *P < 0.05, **P < 0.01, ***P < 0.001 based on Two-Way ANOVA with Bonferroni’s multiple comparison post-test. Interactions: a) F (1,90) = 256.94, p < 0.0001; b) F (1,80) = 641.62, p < 0.0001; c) F (1,28) = 3.99, p = 0.05; d) F (1,26) = 33.23, p < 0.0001; e) F (1,130) = 245.44, p < 0.0001. Data represent mean ± s.e.m. HFD, high fat diet (JPEG 1714 kb) [file 401_2016_1595_MOESM1_ESM.jpg]

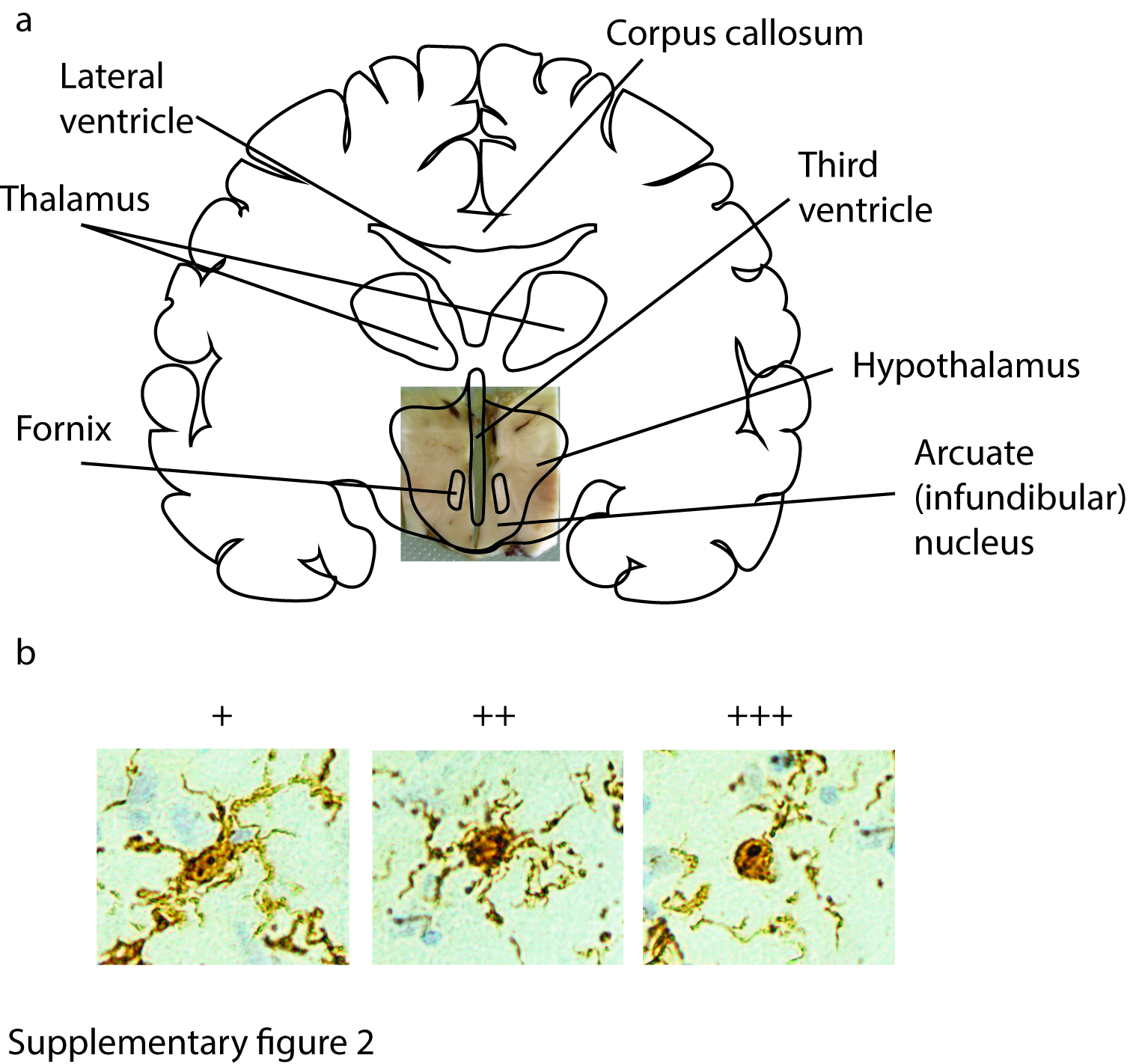

Supplement: Supplementary file 2 — Analysis of human hypothalamus. (a) Illustration of a coronal section of the human brain containing a macroscopic picture of the hypothalamus and neighboring structures. Square indicates the area analyzed for Iba1 and GFAP immunoreactivity. (b) Exemplary images of the three morphological presentations of microglia dystrophy which were defined according to Streit et al. [35]: + beading and partial fragmentation of processes, ++ complete fragmentation of processes while still maintaining cell contours and +++ scattered fragments with intact nucleus (TIFF 10523 kb) [file 401_2016_1595_MOESM2_ESM.tif]
